# Personal Intelligence Is Evident in the Sophistication of People’s Narratives about Personality

**DOI:** 10.3390/jintelligence10030056

**Published:** 2022-08-10

**Authors:** Jayne L. Allen, John D. Mayer

**Affiliations:** Department of Psychology, University of New Hampshire, Durham, NH 03824, USA

**Keywords:** personal intelligence, narratives, personality, sophistication, cognitive complexity

## Abstract

Personal intelligence concerns the ability to understand personality in oneself and others—including the understanding of motives, socioemotional traits, and abilities. We examined if people’s scores on the ability-based *Test of Personal Intelligence (TOPI)* would be reflected in their narratives about someone whose personality they had learned about. In a Preliminary Study (*N* = 220), we collected narratives and open-ended descriptions about their learning. In Study 1 (*N* = 212), experts rated the respondents’ open-ended narratives for their sophistication about personality, defined as their knowledge and complexity of thought around the topic. Respondents also filled out checklists concerning what they learned and their relationship outcomes. Study 2 (*N* = 299) was a replication and extension in which we added the TOPI. Participants who scored higher on the TOPI produced narratives higher in Sophistication, even after statistical controls for Word Count and Vocabulary (the measures also were largely independent of the Big Five). The findings here may have applications for both testing and training.

## 1. Understanding People through Narratives

We use narratives about people to understand their motives and actions in the world (Abell 2004; Schank and Abelson 1977; Sloman and Lagnado 2015; Tomkins 1987). Narratives about the personalities around us help us communicate with one another about who we are and who we believe others to be (Abell 2004; Dunn 2017). In humankind’s era of evolutionary adaptation, accurate personality descriptions allowed group members to detect and understand differences among themselves (Buss 2009) and to keep track of where people were, their activities, and their roles in and contributions to the larger group (Dunbar 2009). Literary critics regard fictional narratives as a form of simulation that can allow us to consider our own and other people’s moral thoughts and acts (Crane 1961). These psychological, evolutionary, and literary viewpoints share the perspective that narrative understandings often convey sophisticated representations of people.

Personal intelligence—the term is parallel to emotional and social intelligences—is a mental ability that involves accurate reasoning about personality in oneself and in other people. It includes the capacities to monitor personality-relevant information, to form accurate models of personality, and to use such information to guide goals and longer-term plans (Bryan and Mayer 2021; Mayer 2008; Mayer et al. 2012). We believe that people high and low in personal intelligence will produce narratives that vary in their sophistication about personality. For example, people higher in personal intelligence should write more knowledgeable, cognitively complex narratives—qualities that can be discerned by attentive observers.

General intelligence surely is reflected in people’s writings. Catharine Cox (Miles 1926) used eminent figures’ letters, stories, and other compositions as youth to demonstrate the differences in thinking of those high and low in intellectual attainment; in so doing, she greatly incremented our understanding of general intelligence and its expression (Simonton 2020, p. 281). Today, general intelligence often is divided into subsidiary and broad intelligences. The widely employed Cattell–Horn–Carroll, or three-stratum model (e.g., McGrew 2009), divides general intelligence (the top stratum) into *broad intelligences* (the second stratum), a group that includes verbal–propositional, spatial, and similar abilities (Carroll 1993; McGrew 2009). These broad intelligences, in turn, help organize and account for people’s abilities to carry out specific kinds of mental tasks (at the third stratum), such as solving vocabulary problems or mental rotation.

The original CHC model focused on the heavily studied intelligences of the 20th century, which chiefly involved reasoning about such things as objects in space, numbers, and word meanings. Since then, however, more people-focused intelligences have been introduced including ability-measured emotional and personal intelligences or have been subject to renewed study, as with social intelligence (Mayer 2018). Recent research indicates that personal, emotional, and social intelligences fit the CHC as part of the broad intelligence level (e.g., subsidiary to *g*) alongside such other instances as spatial, verbal comprehension, and quantitative reasoning (Bryan and Mayer 2020, 2021; MacCann et al. 2014; Schlegel et al. 2019). Personal intelligence is distinct in its focus on reasoning about individual differences in personality (Buss 2009; Christiansen et al. 2005). That reasoning involves understanding the meaning of traits, which traits cooccur, how traits are related to behavior, and such matters as how motives and goals interact among themselves. This focus can be distinguished from that of the ability model of emotional intelligence, which is centrally focused on reasoning about emotions; it is similarly distinct from social intelligence given the latter’s emphasis on social relations (see Mayer et al. 2016, p. 7, for more detail).

### 1.1. The Test of Personal Intelligence (TOPI)

The *Test of Personal Intelligence* or *TOPI* is an ability-based measure of personal intelligence available in several versions (Mayer et al. 2012, 2019). All TOPI versions consist of multiple-choice items about personal intelligence including understanding traits, goals, motives, and similar reasoning. Each test item has a single correct answer scored “1” and three distractors scored “0”, with correct answers keyed to well-established findings in personality research. The test items assess whether people can, for example, understand that liveliness and extraversion go together and identify consistent versus conflicting personal goals (Emmons and King 1988; Goldberg 1993; Goldberg and Rosolack 1994; Mayer et al. 2012, 2019). Personal intelligence appears chiefly unifactorial, with its measure yielding one score to reflect overall reasoning in the area, although highly correlated subfactors cannot be ruled out (Mayer et al. 2019).

In a study of two consecutive classes at the U.S. Military Academy at West Point, cadets with higher scores on the *Test of Personal Intelligence* exhibited higher GPAs in liberal arts courses, such as literature, (for which understanding personality is useful) but less so in STEM courses, even after controlling for other intelligences (Mayer and Skimmyhorn 2017). People higher in personal intelligence also appear more conscientious and open on the Big Five (Mayer et al. 2012, 2018). High scorers report fewer psychiatric symptoms of narcissism and dependent personality disorders, and they engage in fewer counterproductive behaviors at work than others, even after controlling for verbal intelligence (Mayer et al. 2018).

### 1.2. People’s Skill at Describing Personality

People high and low in personal intelligence also vary in how they express their understanding of people. Mayer and colleagues studied ten well-known public figures from Oprah Winfrey to Steven Jobs by collecting observer descriptions, biographies, and related writings concerning their understanding of personality (Mayer et al. 2010; cf., Schultz 2005). These materials then were compiled into individual “biographical record sheets”. Raters were able to reliably identify differences in the leaders’ reasoning about personality from the biographical matter.

More recently, Kenney et al. (2021) recorded the conversations of 135 mother–child dyads, in which the mothers had been asked to discuss the personalities of friends or relatives with their children; those transcripts then were coded for such features as the mention of traits and trait-relevant behavior (Kenney et al. 2021, p. 7). The mothers also took a brief form of the Test of Personal Intelligence to assess their personal intelligence. When asked to compare two people’s personalities, one child of a high-PI mother readily shared that their grandfather “just wants to keep everything clean”, but their grandmother was “…like, ‘Okay go ahead make the mess.’” A child of a lower-PI mother found the task more challenging, concluding about two friends that “Their hair [is what is different]…just different hair color” (Kenney et al. 2021, p. 10). Higher maternal personal intelligence was correlated with personality talk of both the mothers and children, even after controlling for elaborative talk and word count.

### 1.3. Sophistication of Personality Perception

The above findings suggest that aspects of personal intelligence may be discernable in adults’ narratives about themselves and other people. That is, people higher in personal intelligence may communicate more sophistication—more knowledge and complex cognition of personality—in their accounts than those lower in the ability. We expect the empirical relationship to be modest because a person may express themselves well even if what they are saying is not always correct, and different observers will not always agree as to what sounds sophisticated. Sophistication in such narratives would entail the greater use of personality language including trait, goals, and trait-related behavior, embedded in a cognitively complex, meaningful description of people. Cognitive complexity (also called integrative complexity; see Suedfeld and Tetlock 2014) would be reflected in relatively differentiated and integrated cognitive constructs that allow the individual to make finer distinctions, use more relevant-sounding language, and thereby to appear more knowledgeable about a topic (Bieri 1955; Suedfeld and Tetlock 2014). Observed cognitive complexity, although conceptualized as distinct from general intelligence, is also correlated with *g* (Colzato et al. 2006).

### 1.4. Overview of the Present Studies

Will people who score higher on ability-based personal intelligence, relative to those who score lower, write narratives about other people that are higher in sophistication? And, are such narratives related to actual relationship outcomes? To address these questions, we asked our study participants to describe a time they learned about someone and describe the consequences of such learning. We expected key relations between sophistication and personal intelligence to be around *r* = .15 to .25. To test the hypothesis in Study 2, we sought samples near *N* = 200 or greater. (A sample size of 220 would be sufficient to detect *r* = .20 at the *p* = .05 level 85% of the time).

In the Preliminary Study, we developed a survey that asked about a time a participant learned about someone else, a rater-coding system for sophistication based on the narratives, and an open-ended question about the outcome of the relationship. In Study 1, we collected new narratives of learning of the same type, added a relationship-outcome scale based on responses to the Preliminary Study, and examined sophistication and its correlation with other personality measures. In Study 2, we replicated and extended findings from Study 1, adding in an ability-based measure of personal intelligence. The scored data from these studies are available in an open-source file (Allen and Mayer 2022b).

### 1.5. Preliminary Study

In the Preliminary Study, *N* = 220, we asked college students to “think back in your life to a time, place, or event when you learned something about someone else’s personality and record it in a sentence or two” and then to describe the event in some detail, what they learned from it, and any change in the relationship. Additional survey questions asked how they used the information they learned.

Based on the open-ended responses, we created checklist items that appeared reasonably comprehensive of the participant responses. We catagorized what the participant learned about the target person using a series of brief statements ranging from “is kind” and “can be counted on to keep a secret” to “does not do what she says she will do.” We also characterized the relationship with such items as an “increased feeling of closeness” to “ceased having a relationship” with the target person. Those categories were then developed into checklists of *What was Learned* and *Relationship Outcomes* for use in Studies 1 and 2. The open-ended nature of the Preliminary Study responses ensured that we collected a wide range of answers as the foundation for future scales. Perhaps most importantly, we also tested a provisional rating scale for judges to use when evaluating the sophistication of the narrative concerning personality. We tested the rating scales (described in greater detail in Study 1) with undergraduate raters with some success.

We briefly note that in the Preliminary Study, most participants reported learning about a friend (56%), followed by a romantic partner (15%), with siblings and parents next most common. Participants reported learning by observing the person (77%), listening to them (55%), and hearing about the target individual from someone else (21%). Finally, our participants reported that the learning affected their relationships, with 65% reporting changing their opinion of the person they learned about, and 39% changing their behavior toward them.

## 2. Study 1

In Study 1, we again asked participants to describe a time they learned about a target person’s personality (see Measures, below) followed by the checklists of what was learned and the consequences that ensued. The first Test of Personal Intelligence versions were still in trials and not yet ready for use at the time of Study 1; those are introduced in Study 2. Study 1 focused on the level of sophistication found in people’s descriptions of learning, on what was learned, and the relations of these measures to personality scales more generally including the Big Five, Vocabulary (as a proxy for verbal intelligence), and Psychological Mindedness. The chief hypotheses were:

**Hypothesis** **1.** *Trained raters who examined the “Learning About a Person” narratives would be able to reliably detect differences in participants’ sophistication about personality.* We tested this by examining interrater reliabilities of their responses on a sophistication measure with seven rating scales.

**Hypothesis** **2.** *Participants would express underlying themes concerning what they observed on the “What Was Learned?” checklist. The “What Was Learned?” checklist consisted of items, such as “was not trustworthy” and “could be depended on”, populated from open-ended responses to the Preliminary Study. We planned to apply factor models to the data to understand if the participants’ endorsements of these items reflected any underlying themes. If successful, we intended to form factor-based scales from the measure.*

**Hypothesis** **3.** *Whatever the participants learned would have consequences for their ongoing relationships. People use their intelligence to solve problems in their lives, including whether to strengthen or sever a relationship. To reflect that key process, we asked participants to complete a Relationship Outcome checklist, reflecting a strengthening, weakening, or termination of their relationship. We hypothesized that one third or more of the participants would feel either closer or more distant from the target person after the learning incident.*

**Hypothesis** **4.** *The Rated Sophistication Scores of the participants’ learning narratives would correlate with higher levels of Psychological Mindedness (psychologizing about oneself and other people), Openness/Intellect of the Big Five, and with Vocabulary all at modest levels, e.g., r = .20 to .30.* We also examined whether Sophistication correlated with Word Count of the narratives as a control variable.

## 3. Methods

### 3.1. Participants

Two hundred eleven students enrolled in lower-level undergraduate psychology classes at the University of New Hampshire (29 men; 183 women, *M_age_* = 19.29 years, *S* = 2.04) participated in the study in exchange for course credit. Participants identified mostly as Caucasian/White (96%) with the next-largest group identifying as Asian/Pacific Islander (2.4%).

**Screening.** After removal of nonrespondents, we conducted checks for overly quick responding, missing data, and longstring responding. Overly quick responding was defined as completing the survey in less than 25 min: e.g., 10 min to complete the narrative and 2 s each for the remaining 471 survey items (cf. Huang et al. 2012). All remaining surveys were retained: No participant took less than 25 min to complete the survey; no participant omitted more than 4 missing responses on the personality scales, and just one participant registered a consecutive string of 13 identical responses on the 44 items of the Big Five Inventory (completed near the end of the survey)—and those 13 were endorsements of the “Neither agree nor disagree” response (Johnson 2005).

### 3.2. Measures

**Demographics.** Participants were asked their age, gender, and other demographic data.

**Learning about a Person Survey.** The survey consisted of four sections.

***Narrative of learning about a person.*** The narrative portion of the survey asked respondents to “think back in your life to a time, place or event when you learned something about the personality of someone you know well” and then to “describe it in as much detail as you can”, with suggestions to “tell a story, including specific details and feelings” as to what happened. The instructions were followed by nine blank lines to suggest the length the participant might write. A series of checklists then followed.

***Who was the target, and what was the method of learning?*** Participants were asked about their relation to the target (e.g., friend, romantic interest, etc.), how long they had known them, and how the learning took place (e.g., through direct observation).

***What Was Learned Checklist.*** Participants were asked to complete the phrase “I learned this person…” by checking Yes or No to 38 items, such as “was not trustworthy,” “was cruel or mean,” and “was kind,” with the phrasing drawn from responses in the Preliminary Study.

***Relationship Outcome Scale.*** Respondents read the phrase “Because of the event…”, and checked Yes or No to ten potential changes concerning their post-event relationship, from “I ceased having a relationship with this person” to “I feel closer to this person”. An additional five items were removed from the scale before the analyses: two because they referred to behavior of the target person rather than the respondent and the last three items because they were overly broad and were summaries that were redundant relative to the earlier items.

## 4. Sophistication Rating Scale

A scale was prepared for judges to use when rating the sophistication of the narrative portion of the survey. The scale consisted of three items assessing personality content (traits, motives, and development), three items assessing cognitively complexity (balance, empathy, and responsibility), and an “overall impression”. Brief descriptions of the scales can be found on the left-hand side of Table 1.

**Criterion Personality Scales.** Several personality scales were included as criteria.

***Big Five Inventory*.** The 44-item *Big Five Inventory* measures Extraversion, Neuroticism, Conscientiousness, Openness, and Agreeableness (John et al. 1991).

***Psychological Mindedness Scale.*** The 45-item *Psychological Mindedness Scale* assesses a person’s introspective awareness of psychological processes (Conte et al. 1990). Sample items include “I am always curious about the reasons people behave as they do” and “Emotional problems can sometimes make you physically sick”. Respondents answered on a 5-point scale from 1 = “Disagree strongly” to 5 = “Agree strongly.”

***Vocabulary 30 Test.*** The Vocabulary 30 Test (Vocab-30) is a 30-item, multiple-choice measure of American English vocabulary intended as a quick index of verbal intelligence (Mayer and Caruso 2021).

**Other Measures and Scales.** Several other measures were collected but were not reported here because they either were peripheral to our hypotheses, e.g., whether participants generalized what they learned to other people, or because they overlapped with measures we did report, e.g., whether something positive learned about the target was also a perceived cost or a benefit to the respondent. We also attempted to assess the participants’ self-rated sophistication, but the multiple-choice scale we created lacked reliability (*α* = .12) and was dropped. For details, please see the Technical Supplement (Allen and Mayer 2022a).

## 5. Results

### 5.1. Preliminary Analyses

#### 5.1.1. General Criteria for Factor Scales

In general, when creating factor models of our new measures, we sought solutions that fit the data with an RMSEA of .06 or less and a TLI and CFI of near .95 or more (e.g., Hu and Bentler 1999). That said, there were instances where we settled for something near that.

#### 5.1.2. Creating a Factor-Based Index of Relationship Outcome

We applied an exploratory factor analysis to the 10-item Relationship Outcome scale of closeness–distance. A one-factor solution fit the data with an RMSEA = .06 and a CFI and TLI of .999 and .998. Items indicating greater closeness included “I spend or want to spend more time with this person” and “I feel closer to this person.”; those reflecting greater distance included “I feel more distant from this person” and “I ceased having a relationship with this person”. The Relationship Outcome scale was constructed so that higher scores reflected relationship closeness. To do this, the six items representing greater distance were reverse scored. The full scale and factor analyses can be found in the Technical Supplement (Allen and Mayer 2022a).

#### 5.1.3. Descriptive Statistics

**Characterization of the Learning Narratives.** Most of the sample reported they had learned about a friend (63%), followed by a relationship partner (19%), a parent (6%), and a sibling (5%), with lower rates for other family members and authority figures. Most knew the person about three years, but familiarity ranged from one month to all the person’s life. Most of the respondents learned from observing the target’s behavior (90%), including in direct conversation (33%) or watching them speak with someone else (41%). They relied to a lesser extent on hearing from other people (28%). We further examined the narratives so as to identify plots that recurred across narratives and that could be used to briefly communicate their key content: The most frequent plots (which likely overlapped a bit) focused on (a) a secret that was revealed, (b) a betrayal (often romantic), (c) witnessing a single act by the target person that changed how the participant viewed them, and sometimes (d) a more developmental view of gradual change.

**Scale Statistics.** The means, standard deviations, and reliabilities for the central measures can be found in Table 2. Positive Learning is reported as the number of positive items endorsed about the target person; Negative Learning is reported similarly for the number of negative items. For Relationship Outcome, higher scores reflect more closeness. Average scores on the standard personality scales and the scale reliabilities were in line with prior publications.

### 5.2. Tests of Hypotheses

Could Trained Raters Reliably Detect Differences In Participants’ Sophistication? (Hypothesis 1)

To test whether trained raters could reliably detect differences in sophistication across narratives, we enlisted two trained undergraduate judges to evaluate the Learning Narrative and evaluate it on the Sophistication Rating Scale reproduced in Table 1. The scale items are divided between those focused on expressed knowledge about personality (e.g., mention of traits) and those concerned with cognitive complexity in the realm (e.g., “nuanced as to what is good or bad”. A third set of ratings was supplied by a team that consisted of the first author and a third trained undergraduate. Two or more raters responded to each item for nearly all narratives, although owing to external demands on the raters’ time, there were some missing data. Interrater agreement was assessed using a two-way mixed-effect, consistency, and average measures intraclass correlation (Hallgren 2012). This statistic was calculated across raters for each sophistication category, and ICCs ranged from .65 to .77, *M* = .70, indicating good agreement (Cicchetti 1994). Hypothesis 1 was supported. Consistent with earlier studies, our trained raters reached general agreement as to whether the 199 narratives were high or low in sophistication about personality.

**Creation of the Sophistication Scale.** We next created a Sophistication Scale by first taking the average rating across raters for each of the seven scales of Table 2. We tested one- and two-factor models of the seven ratings based on their correlations across the 211 narratives, using exploratory factor analysis employing a maximum likelihood extraction in Mplus (Muthén and Muthén 2017). A one-factor model, with factor loadings shown in Table 2 (right-hand columns) fit the data approximately, with the RMSEA exceeding our criterion at .10, but CFI and TLI meeting criteria at .968 and .952, respectively.

A two-factor model distinguished the two empathic and balance items from the rest, but because the model included a Heywood case (of 1.004) and the two factors were highly correlated at *r* = .83, the one-factor approach seemed better. Further details can be found in the Technical Supplement, Chapter 3 (Allen and Mayer 2022a)

Did Participants’ Conceptions of What They Learned Fall Along Recognizable Themes? (Hypothesis 2)

We next tested whether participants exhibited any coherent themes on the 38-item *What Was Learned Checklist.* Recall this scale contained a diverse set of possible lessons about the target person from “was deceptive or phony” to “could be depended on.” To evaluate the responses, we again applied an exploratory factor analysis in Mplus to the data. The dichotomous responses (Yes–No) were treated as categorical, and a Crawford–Ferguson Facparsim rotation was employed to promote the even distribution of items across factors (e.g., Finch 2011). Two factors were sufficient to fit the data fairly well: *Χ^2^* (628) = 1272.44, *p* < .001; CFI = .95; TLI = .94; and RMSEA = .07 with a correlation between factors of *r* = −.35. We next constrained the solution to a simple structure (i.e., each item loading on just one factor) and removed items that loaded on both factors. The constrained model fit with an RMSEA = .059 and a CFI and TLI both = .98, with 12 items on each scale.

The first Negative Learning factor loaded items such as (the target person) *was not trustworthy*, *could not keep a secret, was cruel or mean,* and *was deceptive or phony*; the Positive Learning factor loaded items such as *could be depended on, was kind, genuinely liked and/or cared about me,* and *was open-minded.* Hypothesis 2, that people’s learning fell into meaningful categories, was supported. Although other models might represent these data equally well (e.g., Mulaik 2005), the two-factor model is parsimonious and has the advantage of representing mixed feelings as might be expressed, for example, if the target were “judgmental” but nonetheless “could be depended on”. Two 12-item scales were created from the results. The fuller analyses are in the Technical Supplement, Chapter 7.

Were There Consequential Changes in the Relationship After Learning? (Hypothesis 3)

To test whether participants reported consequential changes about the relationship after the learning, we examined scores on the overall Relationship Outcome scale (see Preliminary Analyses). Designating “substantial change” in a relationship is admittedly a matter of judgment. For our purposes, we defined substantial change as checking either 60% of the greater closeness items or 60% of the greater distance items. Using this criterion, about 93% of the samples reported a substantial change in their relationship—either closer (50.7%) or more distant (42.2%). Hypothesis 3 also was strongly supported.

Did Sophistication of Learning Correlate with Criterion Scales as Predicted? (Hypothesis 4).

Table 3 reports the correlations among key variables of the study. Sophistication correlated *r* = .10, n.s. with Openness/Intellect, *r* = .13, *p* < .051 with Vocabulary, and *r* = .10, n.s. with Psychological Mindedness, all below predicted levels. Hypothesis 3 was not supported on the whole, with the exception of a marginal relationship of Sophistication with Vocabulary scores.

Sophistication did relate rather strongly to Positive Learning (*r* = .23, *p* < .01), and even more strongly (negatively) to Negative Learning (*r* = −.31) and most of all with increased closeness on the Relationship Outcome measures (*r* = .39, *p* < .001). We further note that Sophistication Scores correlated *r* = .40, *p <* .001 with Word Count. A scatterplot indicated that raters were especially inclined to judge the very shortest narratives as less sophisticated. Upon dropping the 70 participants (33%) who wrote less than 175 words, the correlation between Sophistication and Word Count was reduced to *r* = .14, n.s.

## 6. Study 1 Discussion

Study 1 asked participants to report a learning narrative and included checklists of what was learned about the target individual and of relationship outcomes. Unsurprisingly, Positive Learning led to heightened closeness with the target person on the Relationship Outcome scale (*r* = .72. *p* < .001); Negative Learning led to an increased distance (*r* = −.83, *p* < .001). The Positive and Negative Learning Scales and Relationship Outcomes were mostly unrelated to measures of Vocabulary, Psychological Mindedness, or the Big Five. One exception was that individuals higher in Conscientiousness made fewer claims as to what they learned about the target than others—perhaps reflecting greater circumspection around their perceptiveness.

Raters were able to evaluate the narratives with a high degree of agreement, albeit they tended to perceive less sophistication among the shortest one third of the narratives (Word Counts < 175). People who know more about personality are likely to have more to say about it, and to write at greater length about what they learned. Those who know less would have less to write about. That acknowledged, raters also might have developed the opinion that shorter descriptions were generally lower in sophistication, and this might have influenced their rating behavior to some extent.

People with higher Sophistication Scores also reported more Positive Learning and closer Relationship Outcomes. Contrary to our predictions, however, Sophistication scores only minimally correlated with Vocabulary at *r* = .13, *p* < .051 and nonsignificantly with Psychological Mindedness and Openness, although the relations were in the predicted directions.

The participants in Study 1 readily recalled learning events about people in their lives and had strong emotional reactions to what they learned and different sophistication levels with which they reported such learning. These measures were only minimally related to the Big Five and Psychological Mindedness. We proceeded to explore whether Sophistication Scores might reflect personal intelligence—the capacity to understand personality—in Study 2.

## 7. Study 2

Study 2 replicated and extended Study 1. We examined the stability of our earlier findings and also conducted our key test: Whether personal intelligence, indexed by scores on the *Test of Personal Intelligence* (*TOPI*), was evident in the sophistication with which participants described their learning about personality. Our hypotheses were these:

**Hypothesis** **5.** *Key survey variables would replicate patterns they exhibited in Study 1. That is, Positive and Negative Learning would be negatively related and would be relatively independent of the other variables except for Relationship Outcomes.* Regarding the narrative variables, those with higher Sophistication Scores would write longer (i.e., higher Word Count), at about r = .40, and would report learning more positive qualities about their target person, fewer negative qualities, and feel closer to the target individual than those with lower scores. We further hypothesized that people with higher Sophistication scores would exhibit higher Openness and Vocabulary at about the r = .10 to .15 level.

**Hypothesis** **6.** *Participants would report substantial changes in relationship outcomes after the learning event.* This hypothesis is unchanged from Study 1.

**Hypothesis** **7.** *People’s level of personal intelligence, as assessed by the Test of Personal Intelligence (TOPI), would correlate with the rated sophistication scores of their narratives of learning about the target’s personality.* Our expectation was that TOPI scores would correlate with Sophistication Scores between r = .15 and .30.

**Hypothesis** **8.** *The relation between TOPI and Sophistication Scores would persist after controlling statistically for Word Count (narrative length) and Vocabulary.* We planned to test this using regression models.

***Qualitative Description.*** In addition, we intended to present anonymized examples of narratives from people high and low in personal intelligence and develop a qualitative description of what those narratives were like.

## 8. Methods

### 8.1. Participants

**Sample.** Participants were *N* = 299 college students after screening. Ethnicity was not collected in Study 2, but the sample composition was likely similar to Study 1.

**Screening.** Of the 346 participants who logged into the survey before the cutoff date for completion, 34 were nonrespondent (e.g., almost all items blank), and 13 more were flagged and removed for one or more of the following reasons: More than half their data missing (*n* = 6), no learning episode narrative (*n* = 5), no TOPI responses (*n* = 1), took the survey twice (the second instance was removed) (*n* = 1), signs of inattention on the attention check items because of longstring responding (*n* = 4), or were 17 years old and did not meet the age requirement (*n* = 2). This left the sample of *N* = 299, whose data were analyzed in the next sections.

### 8.2. Measures

**Demographics.** Participants reported their age and gender.

**Learning about a Person Survey.** The same open-ended narrative questions were employed as before. Those were followed by the *Learning about a Person* checklist items. For consistency, we scored the same two 12-item Positive and Negative Learning scales developed in Study 1 (although we had added and deleted several nonincluded items). Following on this was the 10-item *Relationship Outcome Scale,* unchanged.

**Plot classification.** To broadly characterize the contents of the narratives, we asked each participant to indicate which of the several plots we had identified from Study 1 best described their learning event. These were designated by brief names such as “Betrayed!” and “Decisive Act”.

**Sophistication Assessment.** The first author and the same independent raters who evaluated narratives from Study 1 did the same for Study 2, using the same sophistication rating scale developed in Study 1. One rater was not able to complete many ratings, and their evaluations were omitted.

**Test of Personal Intelligence, Version 4.** The Test of Personal Intelligence, Version 4 is a 93-item, multiple-choice, ability-based test of personal intelligence (Mayer et al. 2017, 2019). Each item contains one correct answer scored “1” and three distractors scored “0”. It yields a single overall measure of personal intelligence scored here on an IRT-computed approximation to a T-score scale (Mayer et al. 2017, 2019).

**Big Five Inventory** (John et al. 1991) and **Vocabulary 30 Test.** Both scales were repeated from Study 1.

**Other Measures.** As in Study 1, a group of additional measures were administered but were not analyzed here either because they were overly similar to reported measures, peripheral to the present purpose, or both (see Allen 2017 for details). The scale of Psychological Mindedness from Study 1 was removed owing to its length. Several attention check items were included in the survey and were used for screening.

## 9. Results of Study 2

### Descriptive Statistics

**General Characteristics of the Learning Narratives.** As in Study 1, most of the samples reported they had learned about a friend (61%), followed by a relationship partner (15%), a parent (7%), and a sibling (7%), with lower rates for other family members and authority figures. Most knew the person about 3 to 5 years, but familiarity ranged from one month to all the person’s life. As in Study 1, most of the respondents learned from observing the target’s behavior (85%), including in direct conversation (36%). They relied to a lesser extent on hearing from other people (30%).

We had asked participants to classify their learning event according to one of seven plot descriptions indicated to the left of Table 4 from “A secret revealed” to “Repeated behavior” that we had observed in the narratives of Study 1. To the right, we included very brief excerpts from participants whose narratives conformed to the plot element they rated. For example, in one of the Betrayed! narratives, a participant explained how she was cheated out of being paid back for a loan. Albeit overlap was possible among the plot types, these examples convey a sense of the learning episodes that our respondents described.

## 10. Tests of Hypotheses

Did People with Higher Sophistication Scores Learn More Positive Qualities About the Target and Exhibit Other Relations As In Study 1? (Hypothesis 5)

Key correlations from Study 2 are in Table 5. As in Study 1, higher Sophistication scores were related to Positive Learning (*r* = .13, *p* < .05), Negative Learning (inversely) (*r* = −.25, *p* < .001), and to closer Relationship Outcomes (*r* = .32, *p* < .001). Sophistication scores also correlated with Vocabulary *r* = .25, *p* < .001, more strongly than in Study 1, and still nonsignificantly with Openness at *r* = .11. Sophistication also exhibited a correlation of *r* = .50, *p* < .001 with Word Count. As in Study 1, extremely short narratives were rated lower in personal intelligence. That said, even after removing the shortest narratives (i.e., Word Count < 175), the correlation remained significant at *r* = .33, *p* < .001.

Did Learning About Another Person Change the Status of the Relationship? (Hypothesis 6)

Using the same criteria as Study 2, about 91% of the samples reported a substantial change in their relationship, with 49.8% feeling closer and 40.8% more distant as a consequence of what they had learned—about the same proportions as in Study 1. Hypothesis 6 was supported.

Could Trained Observers Detect Personal Intelligence in Participants’ Narratives of Learning about Another Person? (Hypothesis 7)

A key purpose behind this research was to determine whether independent observers could detect personal intelligence in the narratives our participants wrote. To find out, we correlated the participants’ narrative Sophistication Scores with their TOPI scores and found a relation of *r* = .35, *p* < .001, above our predicted value. Hypothesis 7 was supported.

Did the Relation Between Personal Intelligence and Sophistication Remain After Applying Statistical Controls for Vocabulary and Word Count? (Hypothesis 8)

Given the positive correlations among Vocabulary, Word Count, Sophistication, and Personal Intelligence, one might reasonably wonder if the Sophistication–TOPI relationship would remain after statistical controls for the key related variables. To test this possibility, we regressed TOPI scores against Word Count and Vocabulary in a first model and then added Sophistication in a second model. In the initial model, Vocab-30 and Word Count by themselves predicted the TOPI scores *R* = .52, *F* (2, 296) = 55.67, *p* < .001—a high bar to exceed. Nonetheless, in the second step of the model, the Sophistication ratings incremented the relation with the TOPI to *R* = .57, with an *F_change_* (1, 295) = 22.06, *p* < .001. In the second step of the model, *beta* = .26 for sophistication, with *t* = 4.70, *p* < .001. Hypothesis 8 was supported. Sophistication and Personal Intelligence had their own unique relation apart from the other variables in this study.

Qualitative Differences in the Narratives of People High and Low in Personal Intelligence

Participants in Study 2 provided consent to reproduce deidentified narratives of their learning experiences. To illustrate something of what the raters were seeing, we excerpted three pairs of deidentified narratives in Table 6, each of a person higher and lower in personal intelligence. The reported TOPI scores were converted to an IQ-type scale (M = 100 and S = 15). In Table 6, the higher scorer was 30 or more IQ points above the lower scorer in each comparison. Given that participants who scored higher on personal intelligence wrote more about their learning than those who scored lower (*r* = .22), the pairs of learning narratives were equated for narrative length to illustrate that personal intelligence is not expressed solely as a matter of the number of words employed. Table 6 therefore includes one pair each who wrote short, medium, and long narratives; the specific word counts are recorded at the end of the narrative.

The similarities between people lower and higher in personal intelligence across the narratives were substantial. People lower and higher in personal intelligence both were highly observant of and interested in other people. They both reported a variety of learning experiences from understanding their friends, to leaving relationships, to family conflicts, to falling in love. What they learned could be pleasant or unpleasant. And they both could be either generous or judgmental about the person they were describing.

The chief differences between the two groups appeared in the specificity with which the lower- and higher-ability participants communicated about personalities, the accuracy of their conceptualization (so far as could be judged), the logic of their reasoning, and, maybe, a willingness to consider their own contributions to interpersonal kerfuffles and conflicts.

Differences in specificity are illustrated in Example B, where the lower-PI respondent wrote about her friend M being a “family man” and her perception of “how close knit” the family was after visiting them, but no specifics were provided, and one was left wondering whether the account represented a general impression more than a clearly thought-out response. The paired high-PI respondent, by comparison, is relatively detailed about an argument with her mother, describing both parties as “set in their ways” and how that accounted for the issue. Example C provides an instance of differences in accuracy and logic. The lower-TOPI scorer recounts an ex-boyfriend’s need to be part of the “in-crowd”. Instead of referring to this as status-seeking behavior, she characterizes him as “an easily persuaded human…subject to change with any person’s influence.” The paired high-TOPI scorer more logically depicts the controlling, problematic behavior of a different ex-boyfriend who threatened his friends. Overgeneralization is evident in the lower-PI participant of Example A who remarks “…I would never trust anyone in this world.” The paired high-PI example illustrates the respondents’ willingness to consider their own behavior in interpersonal relations acknowledging, “I am usually late and now I am trying to be more on time…”. More examples of this and additional comparisons can be found in the Technical Supplement, Chapters 8 and 9.

## 11. Discussion of Study 2

In Study 2, we replicated many key findings of Study 1 and found that Sophistication, as evaluated by trained raters, was indeed indicative of personal intelligence measured by the TOPI. In this instance, the relationship remained significant even after controlling statistically for both Word Count and Verbal Intelligence. We further examined qualitative differences of narratives written by those lower and higher in personal intelligence. We consider these findings more fully in the General Discussion.

## 12. General Discussion

Human mental faculties have evolved, in part, to detect individual differences in other people and to use that information to anticipate their behaviors (Buss 2009; Dunbar 2009). People’s narratives about one another help community members to understand and to communicate about the personalities around them. The sophistication of such narratives—from eminent figures’ historical writings to today’s student essays on college admissions exams—have long been associated with the writers’ mental ability (Miles 1926; Simonton 2020).

Personal intelligence can be considered a member of the broad intelligences that make up the current Cattell–Horn–Carroll model of intelligence. Personal intelligence itself is focused on an understanding of mental traits, motives, and goals in oneself and other people. Personal intelligence is fairly independent of spatial intelligence, moderately related to verbal–propositional intelligence, and more closely related—but still conceptually and psychometrically distinct from—emotional and social intelligences (Bryan and Mayer 2021; Schlegel et al. 2019).

Here, we examined people’s narratives of a time they learned about the personality of someone known to them. We wondered: “Would personal intelligence be detectable by a careful and informed reading of such narratives?” We believed that people higher in personal intelligence would express their understanding of personality with greater sophistication, defined as a combination of apparent knowledge of personality (e.g., the use of socioaffective and motivational trait terms) and nuanced cognitively complex judgments (e.g., acknowledging multiple causes of behavior).

We trialed a survey in a preliminary study that asked people about a key time they learned about someone they knew and then asked open-ended questions about the learning event and its consequences. Building on the open-ended responses we obtained, we developed a rating scale for the sophistication of such narratives and checklists concerning what was learned and the outcome of the relationships. These new measures were then deployed in the main studies.

In Studies 1 and 2, we determined, first, that raters exhibited good interjudge reliability for Sophistication when evaluating participants’ narratives across seven rating categories. Those rating categories could be modeled as a single factor, and we constructed an overall Sophistication Score from them that was highly reliable across studies.

We created two additional factor-based scales of Positive and Negative Learning from the “What Was Learned?” scale and a further factor-based Relationship Outcome scale to index whether the relationship became closer or more distant. These scales, too, were reliable across studies.

As might be expected, Positive Learning and Negative Learning were inversely related, with *r*s ≈ −.60 across studies, and the more positive (and less negative) the learning, the closer the relationship became. Negative Learning was especially consequential for reduced closeness (including relationship termination), with *r*s = −.83 and −.84 for Studies 1 and 2. Hence, participants viewed what they learned as key to whether they strengthened, maintained, weakened, or abandoned their relationships.

There were very few notable relationships among the group of Positive Learning, Negative Learning, and Relationship Outcome scores and measures of the Big Five. There was a tendency across studies for Negative Learning to correlate with Neuroticism, *r* = .09, n.s., and .25, *p* < .001 in Studies 1 and 2, with a similar pattern for negative Relationship Outcomes. Intriguingly, people higher in Conscientiousness exhibited less Negative Learning *r* = −.18, *p* < .01, and −.11, n.s., perhaps because they accepted greater responsibility for their part in events.

Sophistication correlated with only one Big Five trait in one study (Agreeableness, *r* = .11, *p* < .05, in Study 2). Vocabulary correlated *r* = .19 and .15 with Openness across studies, *p* < .01 and .05, a commonly found effect (Batey et al. 2010; DeYoung et al. 2014; Furnham et al. 2005). The Test of Personal Intelligence scores correlated *r* = .13, *p* < .05 with Conscientiousness in Study 2, a relation also reported in other studies (Mayer et al. 2012; Mayer and Skimmyhorn 2017) and with Neuroticism, which was unique to Study 2.

The key relation between the Sophistication Scores of people’s narratives and the TOPI was tested in Study 2, where they correlated *r* = .35, *p* < .001, a bit higher than predicted. One concern, however, was the degree to which the relation might be influenced by other factors; for example, TOPI scores correlated *r* = .52 with Vocabulary, a proxy for verbal comprehension intelligence (cf., Bryan and Mayer 2020). In addition, Word Count correlated positively with the variables.

To test whether the TOPI scores and Sophistication were uniquely related, we regressed the TOPI first against Word Count and Vocabulary yielding an *R* = .52. Then, in a second model, we added Sophistication Scores. The relation between Sophistication and the TOPI remained even after controlling for both Vocabulary and Word Count (*R* = .57, with an *F_change_* (1, 295) = 22.06, *p* < .001). Note that this was quite a stringent test that likely removed some genuine variance shared by personal intelligence, sophistication, and word count, given that the people who were more knowledgeable about personality used more words to express themselves.

We went on to present examples of high- and low-PI narratives. There were many similarities across the narratives of those lower and higher in personal intelligence. Both groups wrote about friends, family, and others; both learned positive and negative aspects of their target person, and both could be generous or judgmental. The higher-PI group was chiefly distinguished by their higher levels of specificity concerning traits and motives, more logical conclusions drawn, and more accurate generalizations.

## 13. Limitations

The present studies indicate that an individual’s personal intelligence is related to the sophistication with which they learn about another person, and that such learning impacts people’s relationships. It seems plausible that a better understanding of the target individual would lead to better informed choices about interacting with them. That said, we did not collect data relevant to whether higher personal intelligence led to *better* choices by the participant in regard to the target person, which would require other kinds of studies. Additional limitations were present: The research sample consisted chiefly of emerging adults (e.g., Arnett 2000) at a northeast state university with a student body that is under-representative of people of color. Many, although not all the students, were first-year students and first-year psychology majors. (A Year-in-School X Sophistication one-way ANOVA was nonsignificant (F (4, 293) = 1.53, n.s.). Another limitation was that we did not control statistically for any intelligence measure beyond vocabulary (as a proxy for verbal intelligence) in this study. A broader set of intelligences might be desirable to use in the future. As with most studies, confidence in the findings will be strengthened through replication.

## 14. On the New Findings and Conclusions

Earlier research with the TOPI suggested that high scorers experience smoother social relations and experience more social support (Mayer et al. 2012, 2018; Mayer and Skimmyhorn 2017) and exhibit less counterproductive behavior at work because, we have argued, they better understand and can cope with personalities around them (Mayer et al. 2018).

Here, we found that people with higher TOPI scores, compared to those who scored lower, described their learning about another individual’s personality with greater sophistication—more knowledge of personality and complexity of thought. Such findings further validate the personal intelligence concept by speaking to the *process* behind those smoother relationships found in other studies: A person who can better explain to themselves what someone else is like is more likely to cope well with the other individual. More generally, the narrative accounts studied here provide concrete records of how people understand one another (Crane 1961; Dunbar 2009). The learning descriptions illustrate how people operate intellectually on the symbolic content of personality labels and concepts.

The present findings may generate new ideas for constructing measures of personal intelligence. Teachers and trainers who ask people to produce their own narratives also could use them to help people appraise and develop their own sophistication in learning about people. The development of such skill is a part of adult intelligence and expertise both at home and at work (Christiansen et al. 2005; Ericsson 2007; Von Stumm and Ackerman 2013). Collectively, the findings also connect the capacity to reason about personality with key relationship outcomes. These skills may contribute importantly to the individual’s capacity to navigate their personal and interpersonal worlds.

## Figures and Tables

**Table 1:** The Six Rating Scales for Sophistication and their Factor Loadings on the One-Factor Model ^a^.

Scale Labels and Brief Descriptions of Higher-Score Responses	Factor Loading
Balanced description: *Nuanced throughout as to what is good and bad* or *No judgment.*	.78
Empathy: *Very empathic; feels for person and/or the person’s situation.*	.84
Assignment of responsibility: *Sophisticated balance in attributions to person and situation.*	.85
Developmental influences: *Age or upbringing considered with reasonable connections made.*	.64
Attention to specific traits: *Well-developed connections between traits and behavior.*	.73
Motivation and goals: *Understanding of cooperation and/or conflict among goals and motives.*	.69
Overall Impression: *Good understanding and interpretation of person and unfolding events.*	.88

^a^. Exploratory factor analysis in Mplus with Maximum Likelihood Extraction and Geomin Rotation.

**Table 2:** Descriptive Statistics for the Central Measures of Studies 1 and 2.

		Study 1				Study 2		
		Demographics				Demographics		
Overall *N*		211				299		
Age ^a^	*18 to 21*	*22 to 40*		*Unreported*	*18 to 21*	*22 to 40*		*Unreported*
Count	187	10		14	278	11		10
Gender								
Number of Women		182				241		
Number of Men		29				57		
*Unreported*		0				1		
		Key Variables				Key Variables		
	Mean	Std. Dev.	Possible Range	Rel.	Mean	Std. Dev.	Possible Range	Rel.
Narrative Variables								
Positive Learning	.51	.35	0 to 1 ^b^	.92	.44	.36	0 to 1 ^b^	.85
Negative Learning	.37	.34	0 to 1 ^c^	.91	.38	.33	0 to 1 ^c^	.75
Relationship Outcome	.54	.40	0 to 1 ^d^	.94	.54	.39	0 to 1 ^d^	.94
Narr. Word Count	273.4	192.8	-- ^e^	--	316.7	213.6	-- ^e^	--
Sophistication	2.39	.72	1 to 5	.91	2.57	.61	1 to 5	.90
Personality								
Vocab-30	19.10	4.87	0 to 30	.80	17.69	4.95	0 to 30	.81
TOPI-4	--	--	--	--	47.46	8.76	20 to 80 ^f^	.90
Psych Mindedness	161.94	17.39	45 to 225	.88	--	--	--	--
Big Five								
Extraversion	3.42	.74	1 to 5	.85	3.26	.79	1 to 5	.86
Neuroticism	3.07	.70	1 to 5	.81	3.15	.71	1 to 5	.80
Agreeableness	3.69	.47	1 to 5	.57	3.65	.47	1 to 5	.54
Conscientiousness	3.70	.56	1 to 5	.78	3.63	.60	1 to 5	.77
Openness	3.50	.55	1 to 5	.75	3.40	.58	1 to 5	.77

^a^. The age distributions do not add to 299 because some data were missing or unusable (e.g., a nonrelevant string). ^b^. Proportion of positive learning items endorsed. ^c^. Proportion of negative learning items endorsed. ^d^. Proportion of “closeness” items endorsed and “distance” items *not* endorsed (i.e., reverse-scored). ^e^. No limit was placed on the narratives, but the maximum in Study 1 was 1232 words, and in Study 2, it was 1391. ^f^. The TOPI was scaled on a larger sample to approximate a T-scale (e.g., M = 50 and S = 10); the effective range was about +/−3 S.

**Table 3:** Correlations and Confidence Intervals ^a^ Among the Key Variables of Study 1, *N* = 211.

	Learning Survey Variables			Narr. Variables		Personality Variables	
Learning Survey Variables	Pos. Learng	Neg. Learng	Relationsh. Outcome ^b^	Word Count	Sophistication	Vocab-30	Psych Mindedness
Positive Learning	1.00						
Negative Learning	−.60 ***	1.00					
	[−.68, −.51]						
Rel. Outcome	.72 ***	−.83 ***	1.00				
	[.65, .79]	[−.87, −.78]					
Narrative Variables							
Word Count	−.21 **	.19 **	−.20 **	1.00			
	[−.34, −.06]	[.06, .32]	[−.33, −.07]				
Sophistication	.23 ***	−.31 ***	.39 ***	.40 ***	1.00		
	[.10, .36]	[−.43, −.19]	[.27, .50]	[.29, .51]			
Personality Variables							
Vocab-30	−.16 *	−.06	−.02	.22 **	.13	1.00	
	[−.29, −.03]	[−.20, .07]	[−.15, .13]	[.09, .35]	[−.01, .26]		
Psych Mindedness	−.07	.00	−.02	.14 *	.10	.19 **	1.00
	[−.20, .07]	[−.13, .15]	[.12, .15]	[.01, .07]	[−.05, .22]	[.06, .32]	
Big Five							
Extraversion	.02	.05	−.03	.08	−.01	−.13	.20 **
	[−.12, .15]	[−.09, .18]	[−.16, .10]	[−.06, .22]	[−.13, .15]	[−.26, .01]	[.07, .33]
Neuroticism	−.02	.09	−.05	.08	−.01	.05	−.14 *
	[.12, .15]	[−.05, .22]	[−.19, .09]	[−.06, .22]	[−.15, .13]	[−.18, .09]	[−.27, −.01]
Agreeableness	.04	−.11	.05	−.04	.02	.01	.46 ***
	[−.10, .17]	[−.24, .03]	[−.09, .19]	[−.18, .10]	[−.16, .10]	[−.13, .15]	[.00, .00]
Conscient.	−.12	−.18 **	.08	−.05	.08	.11	.35 ***
	[−.25, .02]	[−.32, −.06]	[−.06, .22]	[−.09, .19]	[−.07, .20]	[.02, .25]	[.23, .46]
Openness	.00	−.08	.05	.02	.10	.19**	.29 ***
	[−.13, .15]	[−.06, .21]	[−.09, .19]	[−.12, 16]	[−.02, .25]	[.06, .32]	[.17, .42]

* *p <* .05; ** *p* < .01; *** *p <* .001; ^a^. 95% percent confidence intervals were estimated by the calculator at https://www.psychometrica.de/correlation.html#confidence (accessed on 20 July 2022); ^b^. Relationship Outcome is scored such that higher scores indicate a closer relationship, and lower scores indicate a more distant or ended relationship.

**Table 4:** Examples of Plots from Study 2 As Identified by Participants.

Plots of the Learning Events	Brief Excerpts of Narratives as Examples
A secret revealed	[While we were traveling my friend] poured her heart out and I learned…her father suffered from a disease called schizophrenia.
Betrayed!	[My best friend at the time] was kind of crazy and a free spirit…[and would] con her friends into doing things. One time she asked me for money…When I asked a few months later if she was planning on paying me back she said she already had.
Growth or change over time	My grandmother…has become increasingly less stable with her condition, lashing out at my father and anyone around her.
Decisive act	I learned that my best friend, R…is not a very honest person. On the first day [of dance class]…R told everyone, including the teacher, that we were cousins…I gave her a confused look and she shot one back that said “just go along with it”…
Intimate caring gesture	During [my grandfather’s] funeral…As I walked down the church behind the casket, I began to sob. My sister grabbed my hand and pulled me close to her…Her strength helped me be strong.
New or conditional trait	My friend O got very mad when I arrived late at his house one day.
Repeated behavior	Throughout high school I thought my friend was the coolest person in the world. One day we were hanging out and he was telling a story, and I realized every story he ever told, he was the hero.

**Table 5:** Correlations and Confidence Intervals ^a^ Among the Key Variables of Study 2, *N* = 299.

	Learning Survey Variables			Narrative Variables		Personality	
Learning Survey Variables	Pos. Learng	Neg. Learng	Relationsh. Outcome	Word Count	Sophistication	Vocab-30	TOPI
Positive Learning	1.00						
Negative Learning	−.59 ***	1.00					
	[−.66, .52]						
Rel. Outcome ^b^	.71 ***	−.85 ***	1.00				
	[.65, .77]	[−0.88, −0.81]					
Narrative Variables							
Word Count	−.09	.14 *	−.11 *	1.00			
	[−.20, .02]	[.03, .25]	[−.22, .002]				
Sophistication	.13 *	−.25 ***	.32 ***	.50 ***	1.00		
	[.02, .24]	[−.36, −.14]	[.22, .42]	[.41, .58]			
Personality Variables							
Vocab-30	−.17 **	−.06	−.08	.33 ***	.25 ***	1.00	
	[−.28, −.06]	[−.17 to .05]	[−0.19, .03]	[.23, .43]	[.14 to .36]		
TOPI-4	−.22 ***	.03	−.06	.22 ***	.35 ***	.52 ***	1.00
	[.11, .33]	[−.08, .14]	[−.17, .05]	[.11, .33]	[.25, .45]	[.43, .60]	
Big Five							
Extraversion	.03	−.02	.001	.06	.02	−.13 *	−.06
	[−.09, .13]	[−.13, .09]	[−.11, .11]	[−.05, .17]	[−.09, .13]	[−.24, −.02]	[−.17 to .05]
Neuroticism	−.13	.25 ***	−.16 **	.07	−.02	.10	.13 *
	[−.24, −.02]	[.14 to .36]	[−.27, −.05]	[−.04, .18]	[−.13, .09]	[−.01, .21]	[−.24, −.02]
Agreeableness	.04	−.11	.09	.04	.11 *	−.03	.07
	[−.07, .15]	[−.22, .003]	[−.02, .20]	[−.07, .15]	[−.00, .22]	[−.14, .08]	[−.04, .18]
Conscient.	−.03	−.11	.03	.10	.05	.08	.13 *
	[−.14, .08]	[−.22, .003]	[−.08, .14]	[−.01, .21]	[−.06, .16]	[−.03, .19]	[−.24 to −.02]
Openness	.04	−.01	.01	.21 ***	.11	.15 *	.02
	[−.07, 0.15]	[−.12, .10]	[−.10, .12]	[.10, .32]	[−.02, .20]	[.04 to .26]	[−.09, .13]

* *p* < .05; ** *p* < .01; *** *p* < .001; ^a^. 95% percent confidence intervals were estimated by the calculator at https://www.psychometrica.de/correlation.html#confidence (accessed on 20 July 2022); ^b^. Relationship Outcome is scored such that higher scores indicate a closer relationship, and lower scores indicate a more distant or ended relationship.

**Table 6:** Deidentified Examples of Narratives of Three Different Lengths from People High and Low in Personal Intelligence in Response to the Narrative Survey Questions *.

Lower in Personal Intelligence	Higher in Personal Intelligence
***Example A, Short Narratives (Less Than 50 Words)***	
[TOPI IQ 58] **[Description?]** I was lied to by my mother about something just so I did not argue with her. I was pissed and then realized if I could not trust my own mother, I would never trust anyone in this world **[Learned from it?]** Yes **[Changed your outlook?]** Yes. *42 Words*	[TOPI IQ 106] **[Description?]** My friend O got very mad when I arrived late at his house one day. **[Learned from it?]** yes because I am usually late and now I am trying to be more on time to things. **[Changed your outlook?]** It has changed me to be more on time to certain events. *45 Words*
***Example B, Medium-Length Narratives (Between 51 and 300 Words)***	
[TOPI IQ 84] **[Description?]** My friend M talked about his family very often which led me to believe that he was a family man. We had talked about me meeting his family for awhile when soon the day finally came. He brought me home and introduced me to his mother, father, sister and brother. The way I saw them all interacting with one another and the way they interacted with me showed me how close knit they were as a family. This made me realize how close M’s family truly was. **[Learned from it?]** This has affected the way I react to others. When in family situations it has helped me realize what a healthy family dynamic really is. This has also translated into my own family life, and dealing with issues regarding my family. **[Changed your outlook?]** This has changed the way I look at human culture. I have never really had a healthy family situation, so this made me see what other families are like. This has helped me deal with some of my own family issues. *169 Words*	[TOPI IQ 116] **[Description?]** As my mom taught me to drive, I continuously got frustrated with how difficult it was to learn how to park. I would ask for help but her suggestions did little to help me. When I expressed how difficult it was for me, she typically would tell me to listen to what she was saying. Although we were both trying, we tended to disagree. So much so that once she left the car while I was trying to park because she was sick of arguing. Through this I discovered we are both very set in our own ways which makes it difficult for us to agree. **[Learned from it?]** with other people, especially when it’s in a manner that may provoke argument. It is important to notice these things so I know when to avoid conflict. **[Changed your outlook?]** It has made me realize how many similarities children have with their parents. I didn’t realize how obvious the traits you inherit are until now. *167 Words*
***Example C, Longer Narratives (More Than 300 Words)***	
[TOPI IQ 76] **[Description?]** My best friend and I have known each other since the 1st grade and she has also known her ex-boyfriend since then. They had broke up at the beginning of senior year of high school due to fighting, and taking different paths in life, she was devastated. They had been together for 3 years and had never liked any other people all throughout the years they spent in school. Many times throughout senior year they tried again. He would begin to care for her again, get what he wanted and then leave again. I had known this person for a majority of my life and I could never begin to understand how he was changing because I did not want to face the fact that he was changing and that he really was not the person he acted like in years prior. The minute they broke up he took it upon himself to become a part of the in-crowd. He did it. He was taken in by them and he immediately began to act like those he was around. That was the time when I finally learned something about him and my friend. I learned that he was an easily persuaded human, and that he was subject to change with any person’s influence, but I also learned that my best friend had a quality that was much desired. She was able to make him that happy, wholehearted person all those years. **[Learned from it?]** Yes, it has made me learn that there is fault in every situation. I thought that they were a fairytale, and it was perfect, but that experience taught me to really take a step back and learn from people before I really take them into my life. **[Changed your outlook?]** Yes, it makes me wonder why humans are so susceptible to change and why we think that we need to win the approval of others in order to feel whole and feel like a part of something. ***326 Words***	[TOPI IQ 129] **[Description?]** This past April I learned something about my ex-boyfriend, A.’s, personality that I had never known before. At the time I learned this information, we had been dating almost three years, and I had been friends with A. for almost four years prior to dating him. For the 7 or so years that I knew him, he always seemed to me, to be a kind, caring, go-with-the-flow person. He could often be opinionated, but I had never seen him be controlling or tempered before. It came to be prom season this year, however, when parties were being planned and suddenly the controlling side of him came barreling through. One of our mutual friends, C., had an already planned party, but A. wanted to have his own since C. left out one of the people A. would have had on his guest list. Therefore, A. tried to set up a competing party and this divided our friend group. He became overly controlling and threatened everybody into agreeing to come to his party instead of C.’s. He got very angry and uncooperative. It was a side if A. that I had never seen before and I didn’t like it. **[Learned from it?]** Yes it has. I broke up with A. after this enlightenment because he threatened me. Since then I have been wary of relationships because I’m afraid of how they will treat me. A. had always seemed so nice, and the hostility came about without any warning that I could sense. I’m now overly worried and conscious that maybe there were signs that I had missed, and if there were then I could just as easily miss them in other people as well. **[Changed your outlook?]** I worry more about how unpredictable people can really be. I never saw this piece of his personality in the almost seven years I knew him, and then all of the sudden and came about and threatened me and my relationship with somebody I had previously cared about. It’s concerning to me that humans and their moods/mannerisms can change so fast with little warning. ***342 Words***

* The three narrative survey questions were (1) (abridged) Think about a time, place or event when you learned about the personality of someone you knew well and describe it. (2) What did you learn from it? And (3) Did it change how you think about human nature?

## Data Availability

An open-source data file that contains the quantitative, scored psychological measures reported in these studies are available online at (URL forthcoming). Excepting the deidentified excerpts reported in this article, the remaining narratives are not available: The detail common to a number of entries raised our concern that at least some entries might be potentially identifying, and we sought to ensure the privacy of the participants.
